# A Comparative Study on Optofluidic Fenton Microreactors Integrated with Fe-Based Materials for Water Treatment

**DOI:** 10.3390/mi13071125

**Published:** 2022-07-16

**Authors:** Lijun Liu, Ning Wang, Liang Wan, Chao Zhao, Kunpeng Niu, Dajuan Lyu, Zhaolong Liao, Biao Shui

**Affiliations:** 1 National Engineering Research Center of Fiber Optic Sensing Technology and Networks, Wuhan University of Technology, Wuhan 430070, China; 300512@whut.edu.cn (L.L.); 303642@whut.edu.cn (C.Z.); kunpengniu18@whut.edu.cn (K.N.); 2 State Key Laboratory of Optical Fiber and Cable Manufacture Technology, Yangtze Optical Fiber and Cable Joint Stock Limited Company, Wuhan 430070, China; lvdajuan@yofc.com (D.L.); liaozhaolong@yofc.com (Z.L.); shuibiao@yofc.com (B.S.)

**Keywords:** microreactor, Fe-based sheet, Fenton reaction, MB degradation, reusability

## Abstract

The catalysts employed in catalytic reactors greatly affect the reaction efficiency of the reaction system and the reactor’s performance. This work presents a rapid comparative study on three kinds of Fe-based materials integrated into an optofluidic Fenton reactor for water treatment. The Fe-based sheets (FeSiB, FeNbCuSiB, and FeNi) were respectively implanted into the reaction chamber to degrade the organic dyes with the assistance of H_2_O_2_. In the experiment, by adjusting the hydrogen peroxide concentration, flow rate, and light irradiation, the applicable conditions of the Fe-based materials for the dye degradation could be evaluated quickly to explore the optimal design of the Fenton reaction system. The results indicated that FeNi (1j85) exhibits excellent degradability in the microreactor, the reaction rate can reach 23.4%/s at the flow rate of 330 μL/min, but its weak corrosion resistance was definitely demonstrated. Although the initial degradability of the microreactor by using FeNbCuSiB (1k107) was not as good as that of 1j85, it increased after being reused several times instead, and the degradation efficiency reached >98% after being reused five times. However, the FeSiB (1k101) material shows the worst degradability and recycling. Therefore, in contrast, 1k107 has the greatest potential to be used in Fenton reactors for practical water treatment.

## 1. Introduction

Due to the merits of the precise regulation of fluid and reaction pathway, large surface volume ratio, and easy integration, microfluidic reactors are conducive to accelerating the chemical reactions [1,2,3] and can be used to modify the reaction system for guiding the design of large-scale reactors [4]. More recently, different microfluidic catalytic reactors have been reported with higher reaction efficiency compared to traditional reactors [5,6,7]. In our previous studies [8,9], several planar photocatalytic microreactors have been developed to degrade the organic dye in water. By solving the limitations of mass transfer, photon utilization, and oxygen deficiency, the photodegradation efficiency and the performance of microreactors have been continuously improved [10]. Compared with bulk photocatalytic reactors, the reaction rate constant has been improved by more than two orders of magnitude, showing great potential for water treatment [11].

However, because semiconductor photocatalytic materials (TiO_2_, C_3_N_4_, BiVO_4_, etc.) have low-light utilization efficiency employed [12,13], photocatalytic reactors with low degradation performance still cannot satisfy the requirements of practical water treatment [14]. Therefore, in addition to the structure design for the reactors, the catalytic materials in the reactor are of significance in determining its reaction efficiency and capacity for water treatment [15,16,17]. In recent years, Fenton reactions have been widely used for high-efficiency water treatment [18,19,20]. Thanks to the reaction between H_2_O_2_ and Fe^2+^ to generate •OH with high redox potential (E_0_ (•OH/H_2_O) = 2.73 V) [21], it can effectively degrade most organic pollutants in wastewater. However, there are many drawbacks to utilizing Fenton reagents (Fe^2+^ and H_2_O_2_) directly in practical catalytic reactors. For example, the concentration of Fe^2+^ should be carefully regulated, and they are difficult to recycle, and iron ions are more likely to form ferric hydroxide precipitation, blocking the reactor easily under neutral and alkaline conditions of the Fenton reactions, which also leads to the complex secondary treatment for the iron sludge in practical applications. Recently, some reactors combined with Fe-based materials were reported for water treatment. For example, the Fenton reactors filled with suspended Fe-based powders exhibit a large specific surface area, accelerating the reactions [22]. However, the external equipment for regulating the magnetic field is usually accompanied, and the powder easily agglomerates and leads to reactor blockage. Moreover, the reactors with fixed Fe-based films in the reaction chamber were developed to avoid post-treatment. However, these irreplaceable, fixed iron-based films suffered from corrosion and inevitably degraded after being reused many times [23]. To address the above issues, an optofluidic Fenton reactor is developed to explore the catalytic materials adapted to its water treatment. Three kinds of Fe-based sheets with different crystal structures and surface characteristics are directly implanted and fixed in the reaction chamber, which could also be conveniently replaced. In the experiment, the degradation performance of the devices integrated with different kinds of Fe-based sheets was investigated and compared by degrading methylene blue (MB) in water, especially for its reusability. Their degradability was also optimized by regulating the reaction conditions, such as the hydrogen peroxide concentration, flow rate, and light irradiation, to guide its industrial application.

## 2. Experimental

### 2.1. Device Design

**Figure 1:**
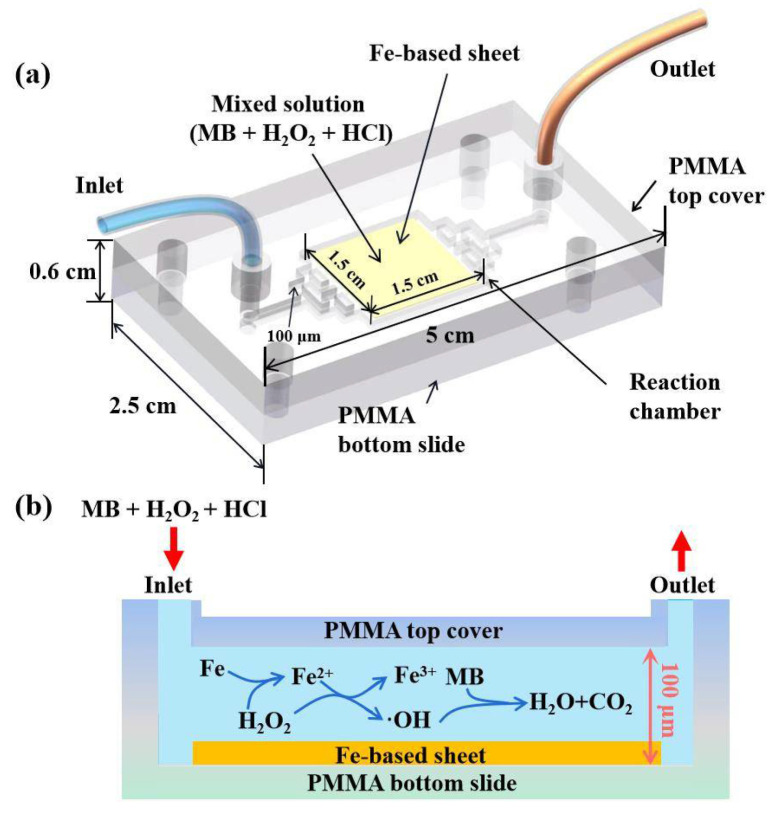
The diagrammatic sketch of the optofluidic Fenton microreactor; (**a**) the 3D structural diagram; (**b**) the cross-section and the reaction pathway.

Figure 1a shows a 3D diagrammatic sketch of the optofluidic Fenton reactor, which is mainly composed of a polymethyl methacrylate (PMMA) top cover, with the microstructures carved by a CO_2_ laser [24] and a PMMA bottom slide. The Fe-based sheet, 15 mm × 15 mm (L × W), was implanted into the rectangular reaction chamber with the dimension of 15 mm × 15 mm × 0.1 mm (L × W × H). The tree-branch-shaped microchannels connecting it with the inlet and outlet were designed to ensure a uniform flow into the reaction chamber. The two pieces of PMMA slides were joined by screws at the corners.

Figure 1b exhibits the cross-section of the Fenton microreactor. The mixed solution of MB, H_2_O_2_, and HCl was first prepared and injected immediately into the reactor from the inlet by a syringe pump, and it flowed evenly into the reaction chamber and reacted with the Fe-based sheet. The reacted solution flowed out from the outlet and was collected by a cuvette to be measured. Here the main reactions that occurred in the reaction chamber could be expressed as follows [25,26]. In short, Fe on the surface of the Fe-based sheet becomes Fe ions and enters into the solution to produce •OH and degrade MB with the assistance of H_2_O_2_.

Fe^0^ + H_2_O_2_→Fe^2+^ + 2OH^−^ (1)

Fe^2+^ + H_2_O_2_→Fe^3+^ + OH^−^ + •OH (2)

Fe^3+^ + H_2_O_2_→Fe^3+^ + •HO_2_ + H^+^ (3)

Fe^3+^ + •HO_2_→Fe^2+^ + O_2_ + H^+^ (4)

H_2_O_2_ + •OH→•HO_2_ + H_2_O (5)

### 2.2. Material and Instruments

Hydrogen peroxide solution (H_2_O_2,_ 30%), hydrochloric acid (HCl, 37%), and Methylene blue (0.05 wt%, MB) were purchased from Sinopharm Chemical Reagent Co., Ltd. All of the reagents were used without any further purification. The 1k101, 1k107, and 1j85 sheets were purchased from Dongguan Chang’an Tongheng Metal Material Company and then cut into squares with the size of 1.5 cm × 1.5 cm with a thickness of 20 μm, 20 μm, and 40 μm, respectively.

A peristaltic pump (LongerPump LSP02-1B) was used to inject the solution into the reacting chamber. The concentration changes of the MB samples were measured by a UV-Visible spectrophotometer (AvaSpec-ABS). Portable UV lamps (emission at 365 nm and 254 nm, ZF-7B and ZF-7C) were employed to irradiate the Fe-based sheet to active the photo-Fenton reactions. The standard CO_2_ laser engraving method was used to fabricate the reaction chamber and microchannels on the PMMA slide.

When preparing the H_2_O_2_ solution with different contents, 90 μL, 150 μL, 210 μL, 270 μL, and 330 μL of H_2_O_2_ were collected, respectively, and added to deionized (DI) water and diluted to 1 mL. To regulate the pH condition in the reaction, 84 μL of an HCl solution (11.9 mol/L) was added to DI water and diluted to 10 mL to obtain the HCl solution (pH = 1). The MB solution (3 × 10^−5^ mol/L) was prepared by dissolving 1 mL of MB solution (0.05 wt%) into 49 mL of DI water. Then the above prepared MB solution, HCl solution, and H_2_O_2_ were mixed together to obtain the original solution samples.

### 2.3. Characterization

Firstly, X-ray diffraction (XRD, Empyrean) was conducted to investigate the structural characteristics of the Fe-based sheets. An atomic force microscope (AFM, NanoWizard 4 XP) was employed to investigate the changing surface morphology of the Fe-based sheet before and after the Fenton reactions, and their hydrophilicity was also investigated by measuring the contact angles.

### 2.4. On Chip Degradation of MB

To explore the optimal reaction conditions for the microreactors, the concentration of H_2_O_2_, flow rate, and light irradiation were regulated to study their effect on the Fenton reactions and the degradation performance of the microreactors with different Fe-based sheets. To study the reusability of the Fe-based materials in the microreactor, the recycling experiment was conducted at the optimal reaction conditions. The experimental parameters were investigated using operating conditions for the runs as given in Table 1.

According to the Beer–Lambert law, the concentration changes of the MB solution samples are proportional to their absorbance at the maximum characteristic peak of 664 nm, and thus the concentration changes of the degraded MB solution samples could be obtained by:

(6) $$A=lg\frac{I_{0}}{I_{t}}=kbC$$

where *A* is absorbance, *I*_0_ is the incident light intensity, *I_t_* is the transmitted light intensity, and *k* is the molar absorption coefficient of the sample [L/(mol·cm)], a constant when the sample and wavelength are fixed; *b* is the optical path length of the sample (cm, a constant when the spectrophotometer and accessories are fixed); *C* is sample concentration (mol/L).

## 3. Results and Discussions

### 3.1. Characterization

**Figure 2:**
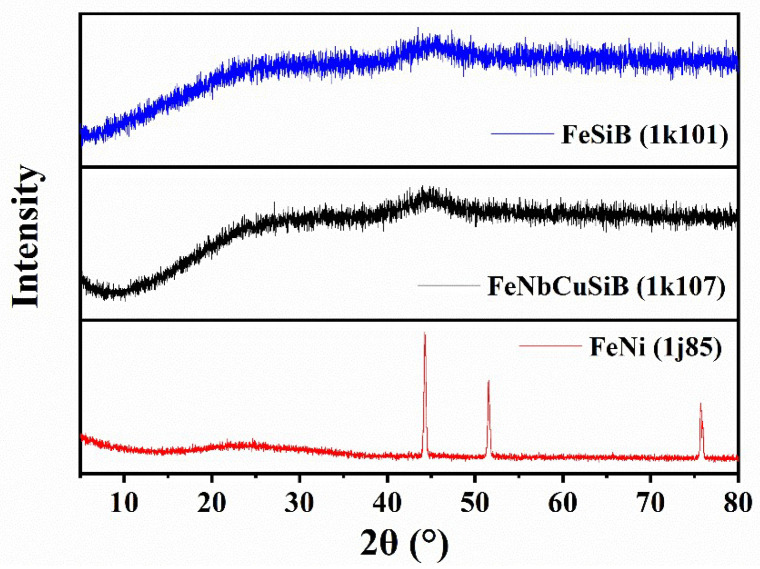
The XRD patterns of the Fe-based sheets.

Figure 2 shows the XRD results of the Fe-based sheets. The blue line represents the XRD pattern of the 1k101, and it can be seen that there is no obvious diffraction peak, indicating the amorphous structure. The black line represents the XRD pattern of the 1k107, and there is a weak diffraction peak at 2θ = 45°, demonstrating its amorphous nanocrystalline structure. The red line represents the XRD pattern of the 1j85, where several obvious diffraction peaks can be observed, showing an obvious crystalline structure in the form of awaruite (PDF# 88-1715, Ni_3_Fe). According to Equations (7) and (8), the adjacent atomic distances (d-space) can be calculated:

(7) $$k=\frac{4\pi sin\theta }{\lambda }$$

(8) $$d=\frac{7.7}{k}$$

where *d* is the adjacent atomic distance (d-space), *λ* is the wavelength of the X-ray source used. Compared to the two amorphous materials, the FeNi alloy has the lowest angle of the maximum diffraction peak, and thus its d-space is the largest, which might cause its corrosion more easily.

### 3.2. Effect of the Flow Rate

**Figure 3:**
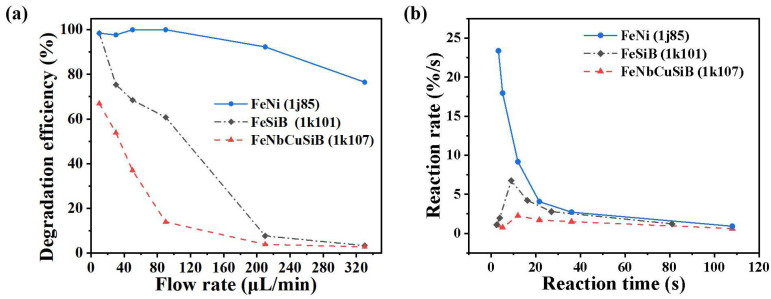
The MB degradation by using the microreactors with the different Fe-based sheets at different flow rates; (**a**) the degradation efficiency; (**b**) the reaction rate as the function of the reaction time.

The flow rate determines the effective reaction time and thus affects the degradation efficiency, and the effective reaction time (residence time) of the MB solution in the reaction chamber could be calculated by:

Reaction time = (Liquid volume in the reaction chamber)/(Flow rate)(9)

where the liquid volume was determined by the reaction chamber volume subtracting the occupied space of the Fe-based sheet in the reaction chamber. To investigate the effect of the flow rate, the concentration of H_2_O_2_ was fixed at a constant of 66 mM, and the solutions were pumped for 10 μL/min, 30 μL/min, 50 μL/min, 90 μL/min, 210 μL/min, and 330 μL/min, respectively. The corresponding reaction time can be calculated by Equation (9) as 108 s, 36 s, 21.6 s, 12 s, 5.14 s, and 3.27 s, respectively, for the microreactors with the 1k107 and lj85, and 81 s, 27 s, 16.2 s, 9 s, 3.86 s, and 2.45 s, respectively, for the microreactor with the 1k101. Figure 3a plots the efficiency of degrading the MB by using the microreactors with the three kinds of Fe-based sheets under different flow rates. It can be seen that as the flow rate increased, the degradation efficiency of all the three microreactors decreased, and when the flow rate was set < 90 μL/min, the degradation efficiency by using the 1j85 remained larger than 97%. One of the reasons for the consistent high degradation efficiency of 1j85 may be that the Fe in the sheet is easier to release from the sheet, and another reason may be the small amount of Mo (~5 wt%) in it. According to previous studies [27], Mo accelerated the reactions dramatically, as follows,

Mo + Fe^3+^→Mo^4+^ + Fe^2+^ (10)

According to Equations (2) and (10), it promotes Fe^3+^ to convert into Fe^2+^ in the solution and produces more •OH participating in the degradation reaction. 

As shown in Figure 3b, the reaction rate when using 1j85 declined rapidly with an increasing reaction time. However, both the reaction rate using 1k101 and 1k107 increased first and then decreased. It is possible that when the residence time was short, it was unfavorable for the reaction referred to in Equation (1), and there was not enough Fe^2+^ in the solution. However, with the increasing reaction time, more Fe^2+^ entered the solution and promoted the reaction process, resulting in an enhanced reaction rate. When the reaction time continued to increase, the content of Fe^2+^ in the solution was relatively sufficient and rapidly converted into Fe^3+^. As reported [26], the reaction in Equation (3) occurred much slower than other reaction processes (*K_3_* = 9.1 × 10^−7^ L·mol^−1^·s^−1^) and thus caused the descending reaction rate. However, the content of Fe^3+^ was always sufficient in this microreactor by using 1j85, and therefore the reaction rate was almost determined by the process in Equation (3).

### 3.3. Effect of the Concentration of H_2_O_2_

**Figure 4:**
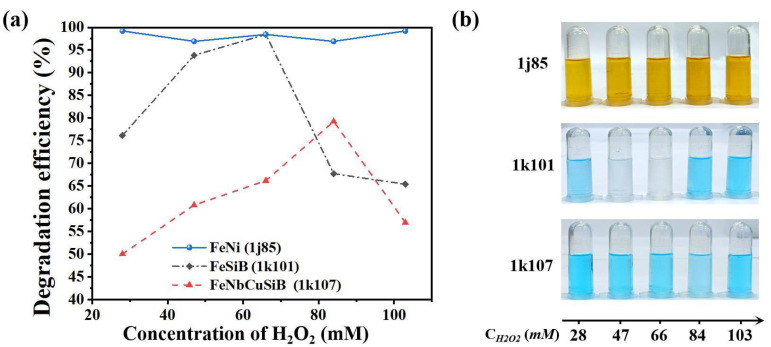
The MB degradation under different concentrations of H_2_O_2_ by using the microreactors with different Fe-based sheets; (**a**) the degradation efficiency; (**b**) The degraded MB solution samples.

According to Equations (1)–(3), the concentration of H_2_O_2_ affects the Fenton reactions significantly by interacting with Fe ion and •OH. To investigate its effect on the degradation performance of the microreactors, the flow rate was fixed as 10 μL/min, and the concentration of H_2_O_2_ was adjusted to 28 mM, 47 mM, 66 mM, 84 mM, and 103 mM, respectively. Figure 4a plots the MB degradation efficiency by using the microreactors with different Fe-based sheets by adjusting the H_2_O_2_ concentration in the MB solution samples. The solid blue line represents the degradation efficiency by using 1j85. It can be seen that when the concentration of H_2_O_2_ varied, the degradation efficiency remained larger than 98%. The black dash-dot line exhibits the degradation efficiency by using the 1k101, which increased first and then decreased, and the optimal degradation efficiency was obtained to be 98.4% at the H_2_O_2_ concentration of 66 mM. The red dotted line shows the degradation efficiency by using the 1k107. The changing law was the same as that of the 1k101, but the highest degradation efficiency was obtained as 79.2% at an H_2_O_2_ concentration of 84 mM. 

As seen from Equations (1)–(3), less •OH was produced at a lower H_2_O_2_ concentration, resulting in declined degradation efficiency. However, it will absorb •OH in the reaction when too much H_2_O_2_ is added to the solution, which reduces the degradation efficiency (Equation (5)). Therefore, there is an optimal H_2_O_2_ concentration for the Fenton reaction in the microreactors. As seen from Figure 4a, the optimal H_2_O_2_ concentration for the reactions using 1k107 was larger than that of 1k101; it can also be speculated that Fe ions were more easily released from 1k101 which will be discussed in detail in the next section. 

Figure 4b exhibits the degraded MB samples under different H_2_O_2_ concentrations by implanting different Fe-based sheets into the microreactors. As seen from the upper panel in Figure 4b, the degraded MB solution became orange, which was mainly caused by a large amount of Fe^3+^ released from the 1j85 sheet, and thus led to the rapid degradation.

### 3.4. Reusability of the Microreactors

To investigate the reusability of the microreactors with different Fe-based sheets, the recycling experiment was conducted five times without replacing the sheets in the reaction chamber. Furthermore, the optimal reaction conditions were chosen here. The flow rate was set to 10 μL/min, and the H_2_O_2_ concentration was regulated as 66 mM for 1k101 and 1j85 and 84 mM for 1k107. To measure the mass of Fe entering the solution from the Fe-based sheets after the reaction, the masses of the centrifuge tubes were first weighed, and the post-reaction solution was collected. By adding enough NaOH solution, all of the Fe^2+^ and Fe^3+^ in the solution were converted into the Fe(OH)_3_ precipitation that was to be centrifuged. After washing with DI water and drying, the total mass of the Fe(OH)_3_ was measured, and then the mass of Fe released from the Fe-based sheets was obtained. 

**Figure 5:**
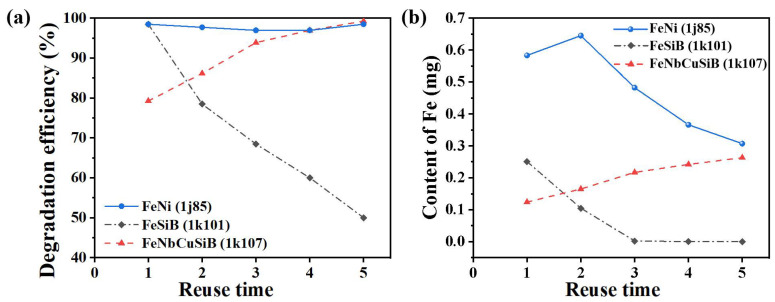
Reusability of the microreactors implanted with the 3 kinds of Fe-based sheets; (**a**) the degradation efficiency of the microreactors reused for 5 times; (**b**) the content of Fe in the reacted solution at different reused times.

The recycling degradation efficiencies of the microreactors are plotted in Figure 5a. The solid blue line represents that of the 1j85 sheet. It can be seen that the degradation efficiency remained more than 95% after being reused five times. The black dash-dot line shows the degradation efficiency of the 1k101 sheet. It is obvious that the degradation efficiency declined gradually from 98.4% to 50% as the reuse time increased. The red dotted line exhibits the degradation efficiency by using the 1k107 sheet. The degradation efficiency rose gradually instead from 79.2% to 99.2% after being reused five times. 

The phenomenon might be explained by the content of Fe in the solution, and thus the reacted solutions were collected to measure that. Figure 5b plots the changing Fe content in the sample solutions when reused at different times. It can be seen that the content of Fe when using the 1k101 Fe-based sheet, became less and less after being reused. However, when using the 1k107 Fe-based sheet, the content increased as the reuse time increased. However, it was also found that the Fe content increased first and then decreased when using the 1j85 Fe-based sheet, and it was always obviously higher than those of the other two Fe-based sheets. Combined with the degradation efficiency in Figure 5a, it can be speculated that the amount of Fe in the reaction should be excessive for the 1j85 sheet. Furthermore, the variation of the Fe content also clearly demonstrated the different degradation efficiency in Figure 5b for the 1k101 and 1k107 sheets.

**Figure 6:**
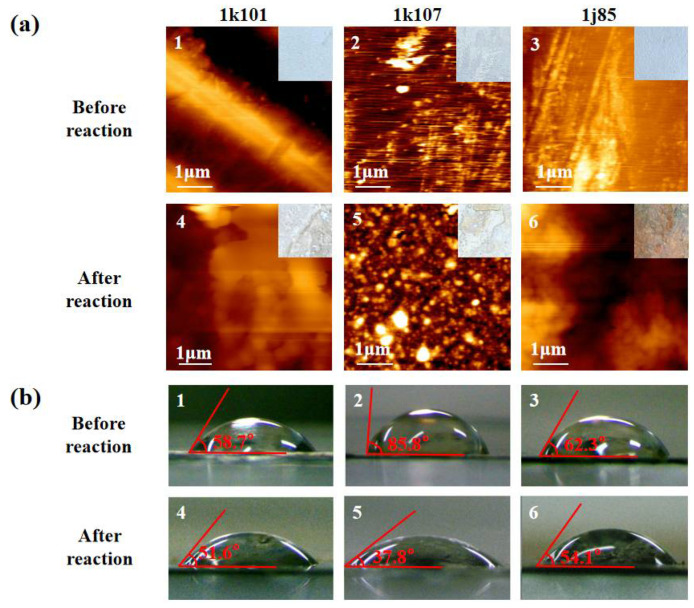
The surface features of the 3 kinds of Fe-based sheets before reaction and after being reused 5 times; (**a**) the AFM photos; (**b**) the images of the contact angles.

To further clarify the reaction kinetics in the microreactors, the surface morphology of the Fe-based sheets before and after being reused five times were first investigated. As shown in Figure 6, it was found that the 1j85 sheet suffered rust corrosion, and no obvious rust was observed on the surface of the other two Fe-based sheets. The surface roughness (RMS) of the 1k101 Fe-based sheet before and after the degradation reactions were measured to be 40.5 and 297.9, respectively, and those of the 1k107 were 2.7 and 14.08, and those of the 1j85 were 15.7 and 246.9. It can be concluded that all the surfaces of the Fe-based sheets became rougher after the Fenton reactions. The 1k101 sheet surface is relatively rougher, and it shows excellent degradability at the beginning. However, an oxide film will gradually form on its surface as the reactions proceed [28], which prevents contact between the solution and the sheet, resulting in significantly decreased Fe content in the solution and declined degradation efficiency. The surface of the 1k107 sheet was smooth before the reaction, and there were relatively fewer active sites on it, resulting in poor degradation. However, its surface became porous, and the solution gradually penetrated into the sheet, then the number of active sites for the Fenton reactions increased a lot. 

The catalytic reactions first occur at the solid–liquid interface between the catalytic material and the reaction reagents, and its surface hydrophobicity will directly affect the contact between the reaction reagent and wastewater samples and then determine the reactor performance. Therefore, the hydrophobicity changes of the Fe-based materials were investigated during their recycle used by measuring their contact angles to optimize the reuse of the reactor. 

The contact angles on the Fe-based sheets before and after being reused five times were also measured, and the images are exhibited in Figure 6b. The initial 1k107 contact angle was 85.8°, while the contact angle of 1k107, after being reused five times, decreased to 37.8°, and there was little change in the contact angle of1k101 and 1j85. It is obvious that the hydrophilicity of the 1k107 sheet was much improved after being reused five times, while those of the other two changed a little. After being reused several times, the ameliorative surface hydrophilicity of the 1k107 sheet improved the contact between it and the solution samples so as to accelerate the Fenton reactions at the solid–liquid surface, which may also be one of the reasons for its enhanced degradability when reused in the microreactors. 

### 3.5. Effect of the Light Irradiation

**Figure 7:**
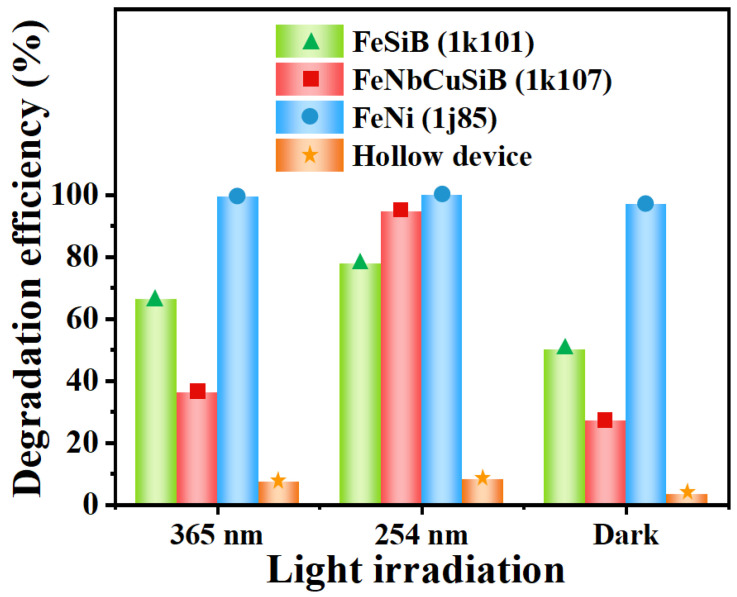
The degradation efficiency of MB by using the microreactors with and without Fe-based sheets under different light irradiation.

To investigate the effect of light irradiation on the photo-Fenton microreactor [28], portable UV lamps with light irradiations of 365 nm and 254 nm were employed to activate the photo-Fenton reactions in the microreactors. Here the flow rate was fixed to be 50 μL/min, and the concentration of H_2_O_2_ was adjusted at the best respective reaction condition (66 mM for 1k101, 1j85 and hollow, 84 mM for 1k107). Considering the effect of light irradiation itself, the control experiment was also conducted, and a hollow microreactor without any Fe-based sheet was used to degrade MB under the same irradiation conditions as above. Figure 7 shows the degradation efficiency under light irradiation and in a dark environment when using the microreactors with and without different Fe-based sheets. The pentagon-marked orange cylinder represents the degradation efficiency when using the hollow device. As seen, only about 3% of MB in the solution was degraded under the dark conditions for the weak effect of H_2_O_2_ interacting with MB directly. Under the irradiation with UV light (365 nm and 254 nm), the degradation efficiency increased a little to 7.62% and 8.23%, respectively. Therefore, the effect of irradiation itself is small and can be ignored. The circle-marked blue cylinder represents the degradation efficiency when using the 1j85 sheet. According to the results shown in the figures and analyses mentioned before, MB can still be almost completely degraded under dark conditions. The triangle-marked green cylinder and the square-marked red cylinder represent the degradation efficiency when using the 1k101 and 1k107 sheets, respectively. It can be seen that UV light irradiation promoted MB degradation, and the performance of the microreactors after being irradiated with the 254 nm UV light source seems better than that of using the 365 nm UV light source.

## 4. Conclusions

In this work, an optofluidic Fenton reaction platform was constructed to study the availability of three kinds of Fe-based materials for water treatment, including 1k101, 1k107, and 1j85. It was found that the microreactors implanted with different Fe-based materials exhibited different features when regulating the H_2_O_2_ concentration, flow rate, and light irradiation. The experimental results show that 1j85 exhibits excellent performance in various reaction conditions and recycling, and the reaction rate can reach 23.4%/s at the flow rate of 330 μL/min. However, serious surface corrosion occurred, and its durability needs to be further verified in practical application. Both 1k101 and 1k107 could obtain enhanced degradability by regulating the reaction conditions in the microreactors, and the former surface will gradually be covered by an oxide layer resulting in dramatically reduced reaction efficiency, which is not suitable for being reused in practical applications. The latter shows enhanced degradability after being reused five times without obvious corrosion, and the degradation efficiency reached up to >95%. Thus, it exhibits the best practicability and is adapted to be used for wastewater treatment.

## Figures and Tables

**Table 1:** Experimental runs and conditions.

Experimental Runs	Flow Rate	Concentration of H_2_O_2_	Light Irradiation
Flow rate	10, 30, 50, 90, 210, 330 μL/min	66 mM	Dark
Concentration of H_2_O_2_	10 μL/min	28, 47, 66, 84, 103 mM	Dark
Reuse time	10 μL/min	66 mM for 1k101 and 1j85, 84 mM for 1k107	Dark
Light irradiation	50 μL/min	66 mM for 1k101 and 1j85, 84 mM for 1k107	Dark, 365 nm UV-A and 254 nm UV-C
